# Exploring the effects of faecal microbiota transplantation on cognitive function: A review of clinical trials

**DOI:** 10.1016/j.bbih.2025.101049

**Published:** 2025-07-04

**Authors:** Sara Alaeddin, Anushka Chatterjee, Tara L. Roberts, Genevieve Z. Steiner-Lim, Slade O. Jensen, Erika Gyengesi, Gerald Muench, Vincent Ho

**Affiliations:** a School of Medicine, Western Sydney University, Penrith NSW, 2751, Australia; b Ingham Institute for Applied Medical Research, Liverpool, NSW, 2170, Australia; c NICM Health Research Institute, Western Sydney University, Penrith NSW, 2751, Australia

**Keywords:** Microbiota-gut-brain-axis, faecal transplant, cognitive function, gut microbiome, Neurodegeneration

## Abstract

Faecal Microbiota Transplantation (FMT) is a widely used microbiota-modulation technique to treat recurrent *Clostridioides difficile* infections (rCDI). Rodent studies and clinical trials on probiotic interventions indicate that alterations in microbiota composition may impact cognitive function. To explore whether FMT influences cognitive function in humans, we conducted a systematic search and narrative synthesis and identified 14 studies examining its effects on cognition. A variety of cohort studies, single-arm trials, case reports and randomised, placebo-controlled trials have been conducted on different neurological patient cohorts, including those with Hepatic Encephalopathy, Parkinson's Disease, dementia, and Mild Cognitive Impairment. FMT has been shown to have a significant impact on cognitive function in these populations, accompanied by alterations in microbial composition and blood markers. Interestingly, success was influenced by the route of FMT administration, indicating greater efficacy of rectal cf. oral administration on microbiome composition and cognitive improvements. However, no clinical trials have yet examined the effects of FMT on cognitively healthy individuals. FMT appears to have potential as a therapeutic strategy for cognitive impairment, though further research with larger sample sizes is needed to explore its effects in both impaired and cognitively healthy populations.

## Highlights

• FMT improves cognitive function in some neurological conditions.
• FMT alters microbiome composition and inflammatory markers in recipients.
• Microbial changes are more pronounced after rectal administration compared to oral.
• Route of administration does not appear to affect the impact on cognition.

## Graphical abstract

Created by Sara Alaeddin in BioRender. Publication licence held by Steiner-Lim, G. (2025) https://BioRender.com/dxzgxui.

**Figure:**
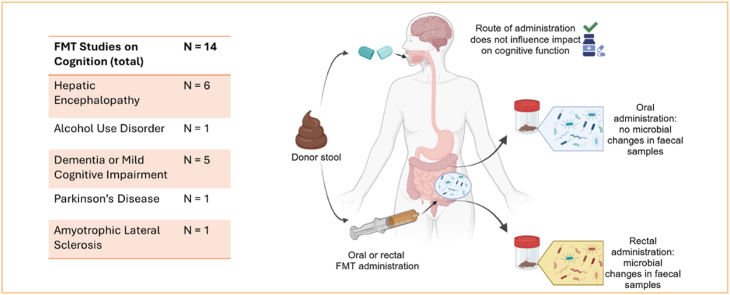

## 1 The gut microbiome and cognitive function

In a world of ever-growing complexity, strong cognitive abilities such as memory, learning and decision-making, are crucial to adapt to new challenges and technologies and to process vast amounts of information. As a result, enhancing cognitive function is a topic of widespread interest. While traditional brain-focused modulation techniques, such as electrical or magnetic stimulation, have been the focus of research for decades, growing evidence suggests that the gut microbiome (hereafter referred to as the “microbiome”) may also play a crucial role in cognitive processes. The microbiome describes the community of microorganisms residing in the gut, including bacteria, archaea, viruses, and fungi (Backhed et al., 2005). Studies on humans suggest an impact of microbiome composition on anatomical brain structure (Fernandez-Real et al., 2015), functional connectivity (Osadchiy et al., 2020), and cognitive function (Anderson et al., 2017).

The microbiome is thought to modulate the gut-brain-axis, a system of multiple, bi-directional pathways enabling communication between the gut and the brain (Cryan et al., 2019) (Fig. 1). Neuroanatomical connections, like the vagus nerve, link the enteric nervous system (encompassing the entire gastrointestinal tract) to the central nervous system and spinal nerve pathways. In addition, the circulatory system allows microbial metabolites to reach the brain (Collins et al., 2012). The microbiome produces metabolites such as short-chain fatty acids (SCFA), secondary bile acids, and tryptophan derivatives (Cryan et al., 2019), which may influence brain function and contribute to neuropsychiatric conditions like Alzheimer's (AD) and, Parkinson's Diseases, and mood disorders (Mirzaei et al., 2021). Furthermore, the microbiome significantly regulates innate and adaptive immune responses (Pieter and Knight, 2014), potentially linking changes in the microbiome to neuroinflammatory and neurodegenerative diseases (Fung et al., 2017). A detailed description of pathways and mechanisms can be found elsewhere (Cryan et al., 2019).

**Fig. 1:**
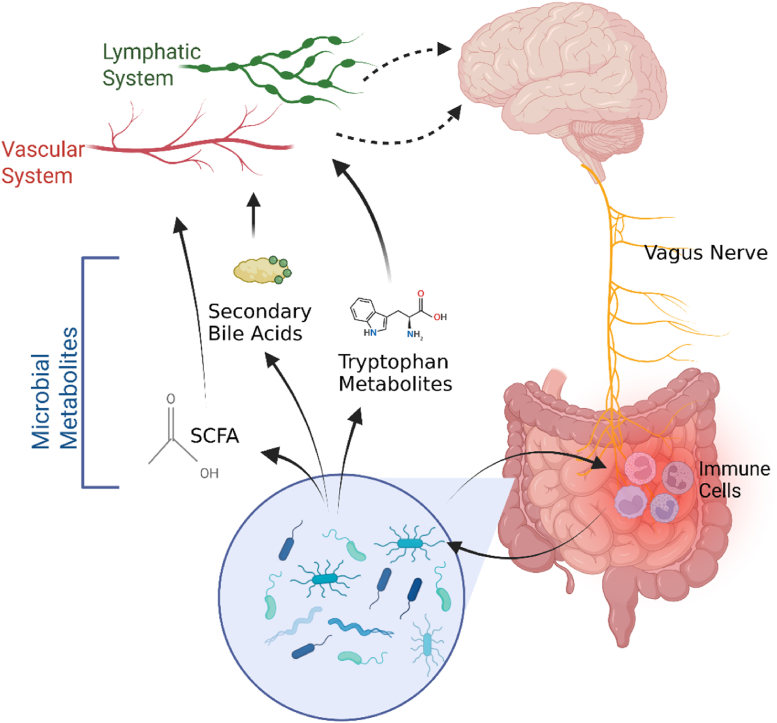
Schematic illustration of the bi-directional pathways of the microbiota-gut-brain axis, facilitating communication between the gut microbiome and the brain, including neuroanatomical routes (e.g. the vagus nerve) and systemic circulation of microbial metabolites. SCFA = short chain fatty acids. Created by Sara Alaeddin in BioRender. Publication licence held by Steiner-Lim, G. Z. (2025) https://BioRender.com/v81y288.

The microbiome composition in infants has been linked to cognitive development, with a higher alpha diversity in infants aged one year old predicting lower visual perception scores and language skills at two years old (Carlson et al., 2018). Interestingly, a lower alpha diversity in healthy older adults has also been linked to poorer cognitive function (Canipe et al., 2021). Moreover, the ratio of bacterial phyla appears to influence different cognitive domains (Manderino et al., 2017). A higher relative proportion of Firmicutes and Verrucomicrobia, compared to Bacteroidetes and Proteobacteria, has been associated with better performance in attention and memory tasks (Manderino et al., 2017). In adults with neurodegenerative diseases, an aberrant microbiome profile has been found, indicating that neurodegenerative diseases are not only associated with a loss of cognitive function, but also a change in microbiota composition (Jemimah et al., 2023). Besides bacteria, the gut virome, which is dominated by bacteriophages, may play a role in executive function and memory (Mayneris-Perxachs et al., 2022), presumably by modulating bacterial composition (Keen and Dantas, 2018). Moreover, gut fungi, also known as the gut mycobiome (Zhang et al., 2021), differ in humans with MCI compared to healthy individuals (Nagpal et al., 2020) and might impact cognitive function through interaction with the host's immune system and the gut microbiome (Enaud et al., 2018). These findings sparked research on microbiome-modulating techniques and their potential impact on cognitive function.

Several studies have explored the effects of probiotics and prebiotics on various cognitive domains in healthy adults. Prebiotics exhibit the potential to improve cognitive function, mental health, and sleep based on available evidence (Paiva et al., 2020). Studies on probiotic use in healthy adults found improvements in memory (Chung et al., 2014), processing speed (Inoue et al., 2018), and brain activation patterns during memory and decision making tasks (Bagga et al., 2018). Adults with cognitive impairments seem to benefit even more from probiotic interventions. A meta-analysis by Zhu, Zhao (Zhu et al., 2021) reported a significant effect of probiotics on cognitive function in adults with MCI, but not in AD. Despite basing their meta-analysis on a subset of five out of the eight studies included in Zhu, Zhao (Zhu et al., 2021)'s analysis, and both meta-analysis incorporating the same studies on AD, Den, Dong (Den et al., 2020) found probiotics to be beneficial in both MCI and AD. Since both analysis included the same AD data, this discrepancy may be due to differences in statistical models utilised, with one applying a more stringent random-effects model (Zhu et al., 2021), which produces more conservative results as opposed to the fixed-effects model (Nikolakopoulou et al., 2014) used in the latter (Den et al., 2020).

In addition to pre-and probiotic supplements, Faecal Microbiota Transplantation (FMT) represents an emerging technique used to alter the microbiome. This review aims to provide a summary of clinical studies investigating the impact of FMT on cognitive function in humans.

## 2 Faecal microbiota Transplantation

FMT involves the transfer of faecal matter from a donor to the gastrointestinal tract of a recipient, either through oral ingestion or rectal administration. This technique, with historical roots tracing back to 400 A.D., was originally used to treat diarrhoea (Wang et al., 2019). Nowadays, its clinical use is indicated for recurrent *Clostridioides difficile* (rCDI) infections that are unresponsive to antibiotic therapy. FMT exhibits an impressive success rate of 83–100 % in the treatment of rCDI, highlighting its ability to induce changes in the microbiome (Seekatz et al., 2014). Despite its success and worldwide use in rCDI, the mode of action of FMT is poorly understood (Hanssen et al., 2021). As an untargeted intervention, FMT transfers a complex mix of donor-derived material, including bacteria, viruses (Zuo et al., 2018a) and fungi (Chen et al., 2023a), unlike probiotics, which introduce only selected bacterial strains. Interestingly, even sterile filtrates of donor stool, i.e. the removal of bacteria and fungi from FMT, has been effective in treating rCDI, suggesting that non-bacterial components of FMT, such as metabolites or bacteriophages, may contribute to its therapeutic effects (Ott et al., 2017). Similar findings in rodent models show that heat-killed probiotics can mimic the cognitive and immune effects of live bacteria, indicating that microbial structural components alone may influence host physiology (Lee et al., 2025). Recent findings also show that bacterial donor strain engraftment in the recipient is not a reliable predictor for clinical improvement (Schmidt et al., 2022). In fact, FMT can introduce novel bacterial strains that were not detectable in either the donor or the recipient prior to treatment (Schmidt et al., 2022). Rather than the engraftment of specific donor-derived strains, donor–recipient microbiome compatibility appears to better explain FMT outcomes. Greater dissimilarity between donor and recipient microbiomes at the strain level, as well as a lower abundance of “gatekeeper” species in the recipient (which can inhibit donor engraftment), are associated with more successful colonialisation, challenging the idea of "super-donors" whose microbiota are universally effective (Schmidt et al., 2022). Furthermore, the donor's gut virome and mycobiome may influence FMT success in rCDI (Zuo et al., 2018a, 2018b). Some studies suggest that increased abundance of SCFA-producing taxa (e.g. *Faecalibacterium*, *Eubacterium*, *Roseburia*, *Bifidobacterium adolescentis*, *B. angulatum*) is associated with better outcomes in ulcerative colitis (Rees et al., 2022) and hepatic encephalopathy (HE) (Bloom et al., 2022). Overall, FMT success seems to rely less on transferring specific taxa or suppressing pathogens, and more on restoring microbial balance (Khoruts and Sadowsky, 2016). Current donor selection guidelines primarily focus on clinical health and infection screening (Cammarota et al., 2019), but emerging evidence suggests that including microbiome compatibility metrics could improve FMT success (Schmidt et al., 2022). Donor age does not appear to be a critical factor for efficacy in rCDI (Anand et al., 2017) and ulcerative colitis, whereas factors such as donor health and microbial diversity seem to play a more important role (Rees et al., 2022).

FMT is currently used in experimental settings only for conditions other than rCDI including Irritable Bowel Syndrome, Inflammatory Bowel Disease, Metabolic Syndrome and neurological disorders such as Autism spectrum disorder, Multiple sclerosis, and Parkinson's Disease, yielding mixed results (Bakker and Nieuwdorp, 2017). Changes in the recipient's microbiome towards the donor's microbiome profile have been found to last for at least 300 days in healthy volunteers (Goloshchapov et al., 2019).

FMT can be administered orally, via nasogastric or -jejunal tube, endoscopy, or encapsulated, or it can be administered rectally via colonoscopy or retention enema (Gulati et al., 2020). Among these methods, colonoscopy and capsules have demonstrated a higher cure rate for rCDI compared to nasogastric administration (Ramai et al., 2021), which may be due to the limited protection nasogastric tubes offer against gastric acid, unlike capsules and rectal administration. Frozen FMT has been found to be as effective as fresh FMT in the treatment of rCDI (Tang et al., 2017).

## 3 FMT on cognition: evidence from clinical trials

To the best of our knowledge, no studies on FMT for cognitive function in healthy human participants have been conducted yet. However, at least one such study is currently underway within our research group (registered under ID ACTRN12623000353695, Australian New Zealand Clinical Trials Registry). Nonetheless, there have been FMT studies in humans from various clinical populations, including neurodegenerative cohorts, where the effects of FMT on cognition function have been explored. Out of 14 studies, five utilised an oral route of administration, while six applied FMT rectally. Given the limited but emerging body of evidence, we conducted a narrative review informed by a systematic search (Turnbull et al., 2023) to summarise current findings on the cognitive effects of FMT in humans. A summary of all trials can be found in Table 1.

**Table 1:** Studies on the effect of FMT on cognitive function in humans.

FMT dose and application	Study population	Study design	Main finding	Cognitive domain	Neuropsychological test	Stool sample analysis	Blood sample analysis	Reference
27g of faeces, administered rectally via enema	Patients with cirrhosis and recurrent Hepatic Encephalopathy (N = 20, 10 FMT, 10 SOC, 20m)	*Randomised, open label.*	Significant cognitive improvement plus beta-diversity changes in FMT group.	Executive function	PHES, EncephalApp Stroop	16S rRNA	–	Bajaj et al., 2017
		Cognition and stool assessed at baseline and day 20						
27g of faeces, administered rectally via enema	Patients with Alcohol Use Disorder (N = 20)	*Randomised, placebo-controlled, double-blind.*	Significant cognitive improvement plus alpha- and beta-diversity changes in FMT group. SCFA in stool increased along with a reduction in IL-6 and LBP in serum.	Executive function	PHES, EncephalApp Stroop	16S rRNA, SCFA via LC-MS	Blood: MELD score, Blood count, Hepatic function, Basic metabolic panel, Serum: IL-6, LBP, Plasma: SCFA	Bajaj et al., 2021
		Cognition, stool, and serum assessed at baseline, day 16 and day 31						
120g of faeces, administered orally via capsules over 5 days	Patients with Hepatic Encephalopathy (N = 10, 6m, 4f, aged 53–72)	*Single arm.*	Significant improvement in PHES scores after FMT. No significant changes in Stroop results, microbiome alpha- or beta-diversity or serum markers	Executive function	PHES, EncephalApp Stroop	Shotgun metagenomic sequencing (using SHOGUN pipeline)	Serum: IL-6, TNF-α, IFNγ, venous ammonia	Bloom et al., 2022
		Cognition, stool, and serum assessed at baseline, after third dose of FMT, 1 week after 5th dose of FMT, and 4 weeks after 5th dose						
30g of faeces, administered orally via capsules over 3 days	Patients with Mild Cognitive Impairment or dementia (N = 5, 3f, 2m, aged 54–80)	*Single arm.*	No significant changes in cognitive scores after FMT. Slight improvement on MoCA in Mild Cognitive Impairment patients. No significant changes in alpha- or beta-diversity or LBP. Changes in serum metabolome (most differentially expressed metabolites after FMT were lipids and lipid molecules).	Screening only: Memory, executive function, visuospatial function	MoCA, ADAS-Cog	16S rRNA	Serum: LBP (ELISA), Serum metabolome (LC-MS)	Chen et al., 2023b
		Cognition, stool, and serum assessed at baseline and days 30, 60, 90 and 180						
150g of faeces, administered orally via capsules over 3 days	Patients with Parkinson's disease (N = 56)	*Randomised, placebo-controlled, single-blind.*	Significant difference between groups in MMSE at week 4 and MoCA at week 12, with greater improvement in FMT group. No significant improvement in alpha- or beta-diversity.	Screenings only: memory, executive function, visuospatial function	MoCA, MMSE	16S rRNA for comparison of FMT, placebo and donor at baseline, shotgun metagenomics for comparing FMT responders to FMT non-responders	–	Cheng et al., 2023
		Cognition and stool assessed at baseline and 4-, 8-, and 12-weeks post intervention.						
1050g faeces administered via colonic transendoscopic enteral tubing over 3∗7 days	Patients with Amyotrophic Lateral Sclerosis (N = 27, aged 18–65)	*Randomised, placebo-controlled, double-blind.*	No difference in cognitive scores after treatment between FMT and placebo group. Significant difference in beta-diversity between FMT and placebo group at week 4 and 12 after treatment. No difference in alpha-diversity or neurofilament light chain protein in plasma.	Screening only: memory, executive function	MMSE	16s rRNA	Plasma: Neurofilament light chain protein	Feng et al., 2024
		Cognition, stool, and serum assessed at baseline and week 4, 12, and 24 post intervention.						
50g of faeces (sterile filtrate), administered orally via nasojejunal tube	Patients with Hepatic Encephalopathy (N = 7, 7m, aged 43–69)	*Single arm.*	No changes in cognitive function. No significant differences in alpha- or beta-diversity. No changes in serum.	Executive function	Number Connection Test (NCT) A, NCT B, digit symbol test	16S rRNA	Serum: Haemoglobin, Leukocytes, Platelets, Aspartate transaminase, Alkaline Phosphatase, Gamma-glutamyl transferase, Bilirubin, Albumin, International normalized ratio, Ammonia, Creatinine, C-reactive protein	Gedgaudas et al., 2023
		Cognition, stool, and serum assessed at baseline and days 7 and 30						
300 ml FMT via colonoscopy	82-year-old male patient with Alzheimer's Disease and rCDI	*Case report.*	Cognitive function improved 2 months after FMT and improved further by 6 months after treatment to a score indicating non-impaired cognitive function.	Screening only: memory, executive function	MMSE	–	–	Hazan, 2020
		Cognition assessed at baseline and 2- and 6-months post FMT						
5.512g of faeces, administered orally via capsules	Patients with cirrhosis and recurrent Hepatic Encephalopathy (N = 20, 20m, 1:1 randomisation to FMT/placebo)	*Randomised, placebo-controlled, single-blind.*	Significant cognitive improvement in Stroop along with significantly lowered LBP levels in FMT group only. Microbiota composition in stool did not differ pre- and post-FMT or between groups. Biopsy pre-and post-FMT showed changes in beta-diversity in duodenum.	Executive function	PHES, EncephalApp Stroop	Stool + upper duodenum biopsy samples analysed with 16S rRNA	Serum: LBP	Bajaj et al., 2019a
		Cognition, stool, and serum assessed at baseline and 3–4 weeks post intervention						
5.512g of faeces, administered orally via capsules	Patients with cirrhosis and recurrent Hepatic Encephalopathy (N = 20, 20m, 1:1 randomisation to FMT/placebo)	*Secondary sample analysis*	Significant reduction of IL-6 in serum and lower primary bile acid levels along with increase of secondary bile acids in blood and stool after FMT but not after placebo.	–	–	16S rRNA, Bile acids: LC-MS	Serum: IL-6, LBP (ELISA), Bile Acids (LC-MS)	Bajaj et al., 2019b Secondary sample analyses of Bajaj et al., 2019a
27g of faeces, administered rectally via enema	Patients with cirrhosis and recurrent Hepatic Encephalopathy (N = 17, 9 FMT, 8 SOC, 17m)	*Long-term follow up of randomised, open-label clinical trial.*	Significant cognitive improvement found in FMT group lasted >1 year, while placebo group still performed at baseline level. Microbiome composition in the FMT group showed changes in beta-diversity that persisted for >1 year.	Executive function	PHES, EncephalApp Stroop	16S rRNA	–	Bajaj et al., 2019c Long-term follow-up of Bajaj et al., 2017
		Cognition and stool assessed 12–15 months post intervention						
60g of faeces, administered rectally via colonoscopy	Patients with Alzheimer's disease and rCDI (N = 5, 3f, 2 m, aged 60–92)	*Single arm*.	Cognitive function improved significantly from pre-FMT to 3 months post-FMT along with significant changes in alpha- and beta-diversity and changes in lipid metabolism gene expression.	Screenings only: memory, executive function, visuospatial function	MoCA, MMSE, CDR, GCS	16S rRNA	Serum: Triglyceride, High density lipoprotein-cholesterol, Low density lipoprotein-cholesterol, Blood urea nitrogen, Creatinine, C-reactive protein, Erythrocyte sedimentation rate, Procalcitonin, Albumin, Lactic acid, Relative gene expression of lipid metabolism (PCR arrays)	Kim et al., 2024
		Cognition, stool, and serum assessed at baseline and 1 and 3 months after FMT						
60g of faeces, administered rectally via enema	90-year-old female patient with Alzheimer's disease and rCDI	*Case report.*	Cognitive scores improved significantly over three months after receiving FMT. Alpha-diversity significantly increased after FMT. No significant changes in beta-diversity.	Screenings only: memory, executive function	MMSE, MoCA, CDR	16S rRNA	–	Park et al., 2021
		Cognition assessed at baseline and 1 and 3 months after FMT. Stool assessed at baseline and 3 weeks after FMT						
60g of faeces, administered rectally via colonoscopy	Patients with dementia and rCDI (N = 20, 16f, 4m)	*Cohort study.*	Cognitive performances significantly increased in FMT group and decreased in antibiotic control group. Significant changes in alpha- and beta-diversity in FMT group only from pre- to post-treatment.	Screenings only: memory, executive function	MMSE, CDR, GCS	16S rRNA	–	Park et al., 2022
	10 FMT vs 10 antibiotics	Cognition assessed 1 at baseline and 1 month after FMT. Stool assessed at baseline and 3 weeks after FMT						

Note. Abbreviations: CDR = Clinical Dementia Rating, GCS = Glasgow Coma Scale, MMSE = Mini Mental State Examination, MoCA = Montreal Cognitive Assessment, ADAS-COG = Alzheimer's Disease Assessment Scale Cognitive Subscale, MELD = Model of Endstage Liver Disease, PHES = Psychometric Hepatic Encephalopathy Score, rCDI = recurrent Clostridioides difficile infection, SCFA = Short Chain Fatty Acids, LC-MS = Liquid Chromatography–Mass Spectrometry, IL-6 = Interleukin-6, TNF-α = Tumour Necrosis Factor alpha, IFNγ = Interferon gamma, LBP = Lipopolysaccharide-Binding Protein.

### 3.1 FMT in cirrhosis and hepatic encephalopathy

Out of 14 studies identified, six investigated the effect of FMT on people with cirrhosis and recurrent HE, i.e. reversible cognitive impairment caused by severe liver disease (Häussinger et al., 2022). The pathophysiology of HE is yet to be fully understood, with evidence pointing towards ammonium neurotoxicity and inflammation (Häussinger et al., 2022). Individuals with HE exhibit significant gut microbial alterations compared to healthy individuals (Bajaj et al., 2012), which may contribute to neuroinflammation (Chen et al., 2020). Clinically, HE presents as impairment in motor skills and cognitive function, ranging from impairments in executive function and processing speed to disorientation and loss of consciousness (Häussinger et al., 2022).

The efficacy of FMT on cognitive impairments in recurrent HE has been investigated in four clinical trials (Bloom et al., 2022; Bajaj et al., 2017, 2019a; Gedgaudas et al., 2023) and further analysed in two additional papers containing either follow-up data (Bajaj et al., 2019c) or a more extensive biomarker analysis of one trial (Bajaj et al., 2019b). All trials included participants with various aetiologies, including alcohol abuse, non-alcoholic steatohepatitis, and Hepatitis C virus. However, none explored the impact of these different aetiologies on outcomes.

Participants showed improvement in executive function after being treated with FMT in single-arm studies (Bloom et al., 2022; Gedgaudas et al., 2023), an open-label trial comparing FMT to a standard of care treatment (Bajaj et al., 2017), and in a single-blind trial comparing FMT to placebo (Bajaj et al., 2019a). Executive function was assessed using the EncephalApp Stroop task, which measures reaction time, and the Psychometric Hepatic Encephalopathy Score (PHES), a standard cognitive impairment measure for HE (Randolph et al., 2009). One study found improvements in both PHES and EncephalApp Stroop scores (Bajaj et al., 2017), while two studies found significant improvements in PHES scores (Bloom et al., 2022) or Stroop scores only (Bajaj et al., 2019a). The fourth study, utilising three PHES-subtests to measure changes in executive function, did not report any significant cognitive improvement (Gedgaudas et al., 2023).

A different trial with a double-blinded, placebo-controlled design, assessed the efficacy of FMT on alcohol cravings and cognitive function in patients with cirrhosis caused by Alcohol Use Disorder, finding an improvement in executive function in the FMT group (Bajaj et al., 2021). Cognitive changes were assessed with the EncephalApp Stroop task and PHES, similar to the studies on HE reported above.

### 3.2 FMT in neurodegeneration

Neurodegenerative diseases entail the progressive loss of neuronal structure and function (Dugger and Dickson, 2017). The underlying pathologies are complex and associated with aggregation of abnormal protein/s (Kovacs, 2019) and dysregulated inflammation (Amor et al., 2010). Aberrant microbiome compositions also occur in various neurodegenerative disorders (Fang et al., 2020), including AD (Jemimah et al., 2023) and Parkinson's disease (Shen et al., 2021). Seven studies have trialled targeting the microbiome in individuals with neurodegenerative diseases via FMT treatment, yielding mixed results (Chen et al., 2023b; Cheng et al., 2023; Feng et al., 2024; Hazan, 2020; Kim et al., 2024; Park et al., 2021, 2022), which might in part be explained by the heterogeneity in conditions, sample size and cognitive assessments.

#### 3.2.1 FMT in motor and movement disorders: Parkinson's disease and ALS

Parkinson's disease is characterised by motoric and cognitive deficits, especially executive dysfunction (Kudlicka et al., 2011), caused by the loss of dopaminergic neurons in the substantia nigra (Dickson, 2018). Interestingly, Parkinson's disease is more likely to occur in individuals with Inflammatory Bowel Disease (Villumsen et al., 2019). As a key regulator of inflammatory cytokines (Schirmer et al., 2016), the microbiome might play a crucial role in the development and/or progression of this neurodegenerative disorder (Nuzum et al., 2022). A single-blind, randomised, placebo-controlled trial in people with Parkinson's disease reported improvements in both MMSE and the Montreal Cognitive Assessment (MoCA), another brief cognitive assessment (Cheng et al., 2023).

Similarly to Parkinson's, amyotrophic lateral sclerosis (ALS) also leads to progressive motor deficits accompanied by executive dysfunction (Mahoney et al., 2021). Despite being characterised as a neurodegenerative disease affecting upper motoneurons, newer evidence points towards more widespread cortical and sub-cortical pathology (Tu et al., 2018).

A double-blind, randomised, placebo-controlled trial in individuals with ALS found no post-intervention improvements in cognitive function in either the FMT or placebo group, as measured by the Mini-Mental State Examination (MMSE), a screening tool for cognitive dysfunction. However, it's important to note that in both, Parkinson's and ALS, executive dysfunction is considered the primary cognitive impairment (Kudlicka et al., 2011; Mahoney et al., 2021). Executive dysfunction refers to impairments in high-level cognitive function such as planning, working memory, mental flexibility, and inhibition (Vasile and Vasiliu, 2021). MoCA and MMSE are brief, general screening tools designed to assess a broad range of cognitive impairments rather than specifically targeting executive dysfunction. More sensitive and specialised assessments, such as the Wisconsin Card Sorting Tests (Vasile and Vasiliu, 2021), which evaluated planning, mental flexibility and problem-solving, may have been able to detect subtle changes in executive function deficits that the MMSE and MoCA may have overlooked.

#### 3.2.2 FMT in Alzheimer's disease and other dementias

Neurodegenerative dementias refer to a group of conditions defined by a decline in two or more cognitive domains, leading to impairments in independent daily life (Gale et al., 2018). Among the most common causes are AD, Lewy Body Dementia and Frontotemporal dementia, all of which are characterised by an accumulation of misfolded proteins and chronic neuroinflammation (Bright et al., 2019; Kinney et al., 2018; Loveland et al., 2023). Emerging evidence suggests that gut microbial changes, such as an increased abundance of pro-inflammatory taxa and a reduction in beneficial bacterial metabolites like short-chain fatty acids, may play a major role in the onset of dementias, particularly in AD and Lewy body dementia (Mateo et al., 2023). Interestingly, differences in microbial composition compared to healthy controls have been observed in adults with MCI (Jemimah et al., 2023), a prodromal stage of dementia characterised by cognitive impairment that does not interfere with the ability to live independently (Sanford, 2017). These findings suggest that microbial alterations may be present before the onset of dementia. Despite the evidence supporting microbial involvement in dementia pathophysiology, few studies investigated the impact of FMT on dementia, with most involving participants with AD (Chen et al., 2023b; Hazan, 2020; Kim et al., 2024; Park et al., 2021), the most prevalent form of dementia (Kinney et al., 2018).

FMT resulted in improved cognitive function in people with AD (Kim et al., 2024). Cognitive scores were assessed one month and three months after FMT and compared to baseline, with an average improvement of six points on both, MoCA and MMSE after three months; a clinically meaningful finding (Lindvall et al., 2024).

A single-arm trial involving people with different variants of dementia or MCI, found an improvement in cognitive function in people with MCI 30 days after FMT; this was further sustained for 180 days after treatment (Chen et al., 2023b). Although people with dementia did not show the same improvements, their cognitive test scores remained at the same level over the six-months re-test period (i.e., they did not decline) (Chen et al., 2023b); possibly suggesting that FMT could help protect against further deterioration in people with dementia. The use of FMT was indicated in both studies to treat an rCDI infection in patients with a neurodegenerative disorder (Chen et al., 2023b; Kim et al., 2024). Significant improvements in MMSE scores in the FMT group were also found in a cohort study that compared people with dementia who were treated with FMT or antibiotics to treat rCDI (Park et al., 2022).

Earlier, two case reports on people with dementia and rCDI, who were treated with FMT, reported significant improvement in cognitive test scores on the MMSE (Hazan, 2020) and MoCA (Park et al., 2021), exceeding the threshold for a clinically meaningful difference in both cases (Andrews et al., 2019; Hensel et al., 2007; Krishnan et al., 2017). These promising outcomes likely inspired the design and implementation of the subsequent single-arm (Chen et al., 2023b; Kim et al., 2024) and cohort studies (Park et al., 2022). All trials on MCI and dementia were conducted with people with rCDI. The rCDI infection itself may have contributed to cognitive impairment, and its cure could account for the observed improvements in cognitive function. However, in one case report, a patient who underwent repeated neurological testing months prior to contracting rCDI consistently demonstrated low scores, which improved only after FMT, suggesting that rCDI was not a confounding factor in that case (Park et al., 2021). Moreover, a cohort study observed cognitive improvements exclusively in the FMT group, with no improvements in the antibiotic treatment group, despite both treatments achieving a 90 % cure rate, which later increased to a 100 % cure rate in the FMT group after a second FMT. These findings suggest that FMT might induce cognitive improvements beyond simply curing rCDI. However, one of the single arm trials (Chen et al., 2023b) reported improvements in MCI, but not in dementia. This might indicate that FMT is more useful in earlier disease stages, similar to probiotics (Zhu et al., 2021). Nonetheless, improvements in MCI could also reflect a practice effect from repeated cognitive testing, a factor less likely to influence individuals with dementia due to significant memory impairments.

Moreover, the available studies on dementia have considerable limitations. In addition to the small sample size, ranging from one to five in all but one study, and the lack of control groups (Chen et al., 2023b; Hazan, 2020; Kim et al., 2024; Park et al., 2021), two studies included participants with different forms of dementia (Chen et al., 2023b; Park et al., 2022). Differences in underlying pathologies and affected cognitive domains add to the heterogeneity, complicating the interpretability of results. This issue is particularly relevant when considering the cognitive assessments used. All studies relied on brief screening tools only, rather than comprehensive neuropsychological evaluation of different cognitive domains. As a result, subtle improvements in specific domains may have gone undetected.

### 3.3 Microbial changes after FMT

Interestingly, microbiome analysis yielded mixed results. Apart from one (Bloom et al., 2022) study basing their analysis on metagenomic shotgun sequencing, all reported studies utilised a 16s-rRNA approach to compare alpha and beta diversity between baseline and follow-up and between groups, if applicable. It is important to note that only metagenomics enables functional profiling of microbial communities and allows for the detection of less abundant genera (Durazzi et al., 2021). In contrast, 16S rRNA analyses may overlook important microbial interactions, functional capacities, or low-abundance species that could be biologically relevant for explaining the mechanisms of FMT (Durazzi et al., 2021). Alpha diversity refers to the diversity of taxa within a single subject's gut, while beta diversity measures the differences in taxa composition between different subjects or time points. Changes in beta-diversity were reported in one out of four clinical trials on HE (Bajaj et al., 2017). These changes were later found to have persisted for over one year (Bajaj et al., 2019c). The double-blind RCT on Alcohol Use Disorder also reported significant changes in alpha- and beta-diversity (Bajaj et al., 2021). Interestingly, both studies found an increase in the relative abundance of *Ruminococcaceae* and other SCFA-producing taxa, such as *Lachnospiraceae*, between pre- and post-FMT. Low levels of *Ruminococcacae* have been linked to cognitive impairment (Hung et al., 2023; Ren et al., 2020).

Three other clinical trials on HE reported no significant changes in microbial diversity derived from faecal samples on a maximum of ten participants each (Bloom et al., 2022; Gedgaudas et al., 2023; Bajaj et al., 2019a). All three trials chose oral administration of FMT, ranging between a total of 120g of stool (Bloom et al., 2022) and 5.5 g of stool (Bajaj et al., 2019a). The latter additionally assessed microbial changes on duodenum-biopsies taken at baseline and follow-up in the FMT-group and found significant changes in the beta-diversity of the duodenum, despite seeing no microbial changes in faeces (Bajaj et al., 2019a). These findings imply that microbial composition changes may go undetected if only faecal samples are analysed.

In clinical trials on FMT in neurodegenerative diseases, six out of seven reported studies assessed microbial changes via 16s-rRNA analysis (Chen et al., 2023b; Cheng et al., 2023; Feng et al., 2024; Kim et al., 2024; Park et al., 2021, 2022). One case study did not analyse microbial samples (Hazan, 2020). Three studies on individuals with dementia found a significant increase in alpha-diversity (Kim et al., 2024; Park et al., 2021, 2022), with two reporting additional changes in beta-diversity (Kim et al., 2024; Park et al., 2022). The study on ALS found changes in beta-diversity only (Feng et al., 2024) four weeks after FMT, with an increase in the relative abundance of *Bifidobacterium*, which was initially higher in donors than in recipients before FMT. *Bifidobacterium* may modulate the gut-brain-axis through the production of the neurotransmitter Gamma-Aminobutyric Acid (Duranti et al., 2020). While gut-derived Gamma-Aminobutyric Acid cannot pass the blood brain barrier, it may indirectly influence cognition by modulating the enteric nervous system and vagus nerve and by modulating inflammation (Chen et al., 2021). Two additional studies on neurodegeneration did not find a significant change in bacterial richness or composition (Chen et al., 2023b; Cheng et al., 2023).

Of the twelve trials assessing microbial changes after FMT, seven observed shifts in alpha and/or beta diversity. Interestingly, these changes did not necessarily result in a microbiome composition closer to that of the donors. In studies that reported changes in alpha diversity post-FMT, all observed an increase in bacterial richness (Bajaj et al., 2021; Kim et al., 2024; Park et al., 2022). Alpha diversity has been shown to correlate positively with cognitive performance in healthy older adults (Canipe et al., 2021), indicating that the change in alpha diversity seen here might lead to improvements in cognitive function. Importantly, all but one study (Bloom et al., 2022) relied on 16S rRNA sequencing, which, unlike metagenomic shotgun sequencing, is limited to identifying bacterial taxa, excluding insights into the virome, fungal communities, and bacterial subspecies (Ranjan et al., 2016). This limitation could mean that significant microbial shifts remained undetected in most studies.

The mechanism of action of FMT is not yet understood. Beyond changes in gut bacterial composition, alterations in bacteriophages, the gut mycobiome, and bacterial metabolites such as SCFA and bile acids have been proposed as contributing factors (Segal et al., 2020). Microbial changes induced by a single dose of FMT demonstrated stability for up to three years (El-Salhy et al., 2022).

### 3.4 Changes in peripheral markers after FMT

Blood samples were analysed in seven clinical trials (Bloom et al., 2022; Bajaj et al., 2019a, 2019b, 2021; Chen et al., 2023b; Feng et al., 2024; Gedgaudas et al., 2023; Kim et al., 2024). One of the markers of interest is lipopolysaccharide (LPS)-binding protein (LBP), an acute phase protein that is produced by the liver and by intestinal epithelial cells (Vreugdenhil et al., 1999). Two studies on cirrhosis found a significant decrease in LBP after FMT (Bajaj et al., 2019a, 2021), while a third study on individuals with MCI and dementia failed to find a change in LBP-levels (Chen et al., 2023b). Regarding inflammatory markers, Interleukin-6, an inflammation-modulating cytokine (Čulić et al., 2001), was significantly decreased post-FMT in patients with HE (Bajaj et al., 2019b) and Alcohol Use Disorder (Bajaj et al., 2021), while another study on HE failed to find any changes in Interleukin-6- or ammonia-levels (Bloom et al., 2022). These findings appear independent of the administration mode, as one HE study used enema (Bajaj et al., 2019c), while the other used oral capsules (Bloom et al., 2022). All studies followed a similar timeframe, with the latest assessments conducted one-month post-intervention. However, each study had only ten FMT-treated participants, with varying underlying causes of HE. The heterogeneity of patients and small sample sizes may explain the differing results. Neurofilament light chain protein as a biomarker for neuronal damage was assessed in one study only (Feng et al., 2024), but failed to show significant changes between FMT and placebo group.

In MCI and dementia, significant alterations in the serum metabolome post-FMT were observed (as assessed via liquid chromatography-mass spectrometry), with the primary affected pathway being bile secretion (Chen et al., 2023b). Strikingly, a study on HE also reported changes in bile acids, with an increase in primary bile acids along with a decrease in secondary bile acids in serum post-FMT (Bajaj et al., 2019b). Secondary bile acids are metabolised by bacteria in the duodenum (Collins et al., 2023), and might therefore serve as a marker for microbial changes. Interestingly, this particular study (Bajaj et al., 2019b) is a secondary sample analysis of a clinical trial on HE in which microbial changes were found in duodenum-biopsy post-FMT, despite not finding changes in faecal samples (Bajaj et al., 2019a). This suggests that serum analysis of bile acid levels may reveal changes in the duodenal microbiome that cannot be detected through faecal analysis. Furthermore, changes in bile acids and SCFAs, which are synthesised or metabolised by the microbiome, have been found to influence cognitive function (Parker et al., 2020). Hence, a trial on people with dementia assessed changes in host gene expression of genes involved in lipid metabolism via PCR on serum and found a continuous upregulation of three genes along with a downregulation of one gene post-FMT (Kim et al., 2024). The lipid metabolism is regulated directly by the gut microbiome, for example, by inhibiting fasting induced adipocyte protein factor and indirectly by producing the SCFA acetate, which promotes lipid oxidation (Jian et al., 2022).

### 3.5 Route of administration

Strikingly, changes in microbial diversity appear to be associated with the route of administration of FMT. FMT can be administered orally, in the form of capsules, through a nasogastric or nasojejunal tube; or rectally, through colonoscopy, enema, or through the emerging technique of colonic transendoscopic enteral tubing (TET). TET enables FMT application to the proximal colon, similar to colonoscopies, but allows for multiple FMT-applications without the need for repeated colonoscopies (Wang et al., 2023).

All studies reporting significant changes in alpha- or beta-diversity derived from 16s-rRNA analysis on faecal samples between pre- and post-FMT chose the lower gastrointestinal tract as a target for delivery, and administered FMT either via rectal retention enema (Bajaj et al., 2017, 2021; Park et al., 2022), via colonoscopy (Kim et al., 2024; Park et al., 2022), or via TET (Feng et al., 2024). However, one study found significant microbial changes in a duodenum-biopsy after delivering FMT orally via capsule (Bajaj et al., 2019a). This might indicate that microbial changes are spatially limited to the proximity of the target location, and alterations in the upper gastrointestinal tract might not be reflected in faecal samples. On the other hand, all but one study used 16S rRNA sequencing, which may have missed changes in non-bacterial components or less abundant genera (Durazzi et al., 2021).

Interestingly, despite different modes of delivery and microbial changes, only two trials failed to show improvements in cognitive function: a study on ALS (Feng et al., 2024) and a study on HE (Gedgaudas et al., 2023). The lack of improvement in ALS may be attributed to the small sample size and the fact that only 20–50 % of individuals with ALS experience cognitive impairment (Mahoney et al., 2021). However, the HE study used a sterile filtrate of FMT, administered via a nasojejunal tube. The filtrate was produced following the protocol by Ott, Waetzig (Ott et al., 2017), who were able to effectively treat rCDI despite removing bacteria from the FMT. This might imply that cognitive changes are in fact driven by changes to the bacterial composition of the microbiome.

## 4 Limitations

Clinical studies on the effect of FMT on cognitive function are rare. Out of eleven clinical trials identified that reported cognitive function as a primary or secondary outcome, only three studies used a randomised, placebo-controlled, double-or single-blinded design (Bajaj et al., 2019a, 2021; Cheng et al., 2023). Of the others, one was open-label (Bajaj et al., 2017), four were single-arm studies (Bloom et al., 2022; Chen et al., 2023b; Gedgaudas et al., 2023; Kim et al., 2024), one cohort study (Park et al., 2022) and two case reports (Hazan, 2020; Park et al., 2021). Moreover, the sample size of each study was relatively small, ranging from five to ten in single-arm trials to a maximum of 56 in a randomised, controlled trial (Cheng et al., 2023). Furthermore, studies on dementia often included participants with different stages or variants (and therefore different pathophysiologies), leading to large heterogeneity regarding cognitive function and disease progression within their sample.

Moreover, clinical trials assess cognitive function in various patient cohorts using a diverse array of cognitive tests and screening tools, making comparisons between studies difficult. Most studies rely on brief cognitive screenings (e.g., MMSE or MoCA) or focus on individual cognitive functions (e.g., via the Stroop Task), potentially overlooking changes in unassessed areas. Future studies should aim at higher sample sizes, less heterogeneity within their sample, and a broader range of cognitive function outcome measures to ensure that no aspect is missed.

## 5 Conclusion

Few studies have explored the effect of FMT on human cognition, with even fewer randomised controlled trials completed. However, research in HE, Parkinson's disease, and dementia suggests that altering gut microbial composition through FMT might enhance cognitive function. Interestingly, microbial changes in faeces appear more measurable when FMT is administered rectally as opposed to oral administration, although this might partly depend on the sample type, as biopsies can reveal greater microbial shifts than stool samples. However, the route of administration or dose does not appear to influence cognitive improvement. The only trial discussed here that did not observe cognitive improvement used a sterile filtrate of FMT, suggesting that bacteria are likely key drivers of cognitive changes. Importantly, FMT-induced cognitive improvements have been shown to persist for up to one year following treatment, highlighting its potential as a convenient and effective long-term treatment option.

## CRediT authorship contribution statement

**Sara Alaeddin:** Writing – original draft, Conceptualization, Project administration, Methodology, Visualization. **Anushka Chatterjee:** Writing – review & editing. **Tara L. Roberts:** Writing – review & editing, Conceptualization, Supervision. **Genevieve Z. Steiner-Lim:** Supervision, Conceptualization, Writing – review & editing. **Slade O. Jensen:** Writing – review & editing, Supervision. **Erika Gyengesi:** Supervision, Writing – review & editing. **Gerald Muench:** Writing – review & editing, Supervision. **Vincent Ho:** Supervision, Writing – review & editing.

## Funding

We acknowledge the support of the Ainsworth Medical Research Innovation Fund, School of Medicine, 10.13039/501100018822Western Sydney University, Australia. GZS's contribution was supported a 10.13039/501100000925National Health and Medical Research Council (10.13039/501100000925NHMRC) Investigator Grant (APP1195709). TLR was supported by an Irene and Arnold Vitocco Research Fellowship.

## Declaration of competing interest

The authors declare the following financial interests/personal relationships which may be considered as potential competing interests: Genevieve Z. Steiner-Lim reports financial support was provided by 10.13039/501100000925National Health and Medical Research Council (10.13039/501100000925NHMRC) Investigator Grant (APP1195709). Tara L. Roberts reports financial support was provided by Irene and Arnold Vitocco Research Fellowship. Sara Alaeddin reports financial support was provided by 10.13039/100014467South Western Sydney Local Health District, Indena and 10.13039/501100018822Western Sydney University Postgraduate Research Scholarship. If there are other authors, they declare that they have no known competing financial interests or personal relationships that could have appeared to influence the work reported in this paper.

## Figures and Tables

**Fig. 1:**
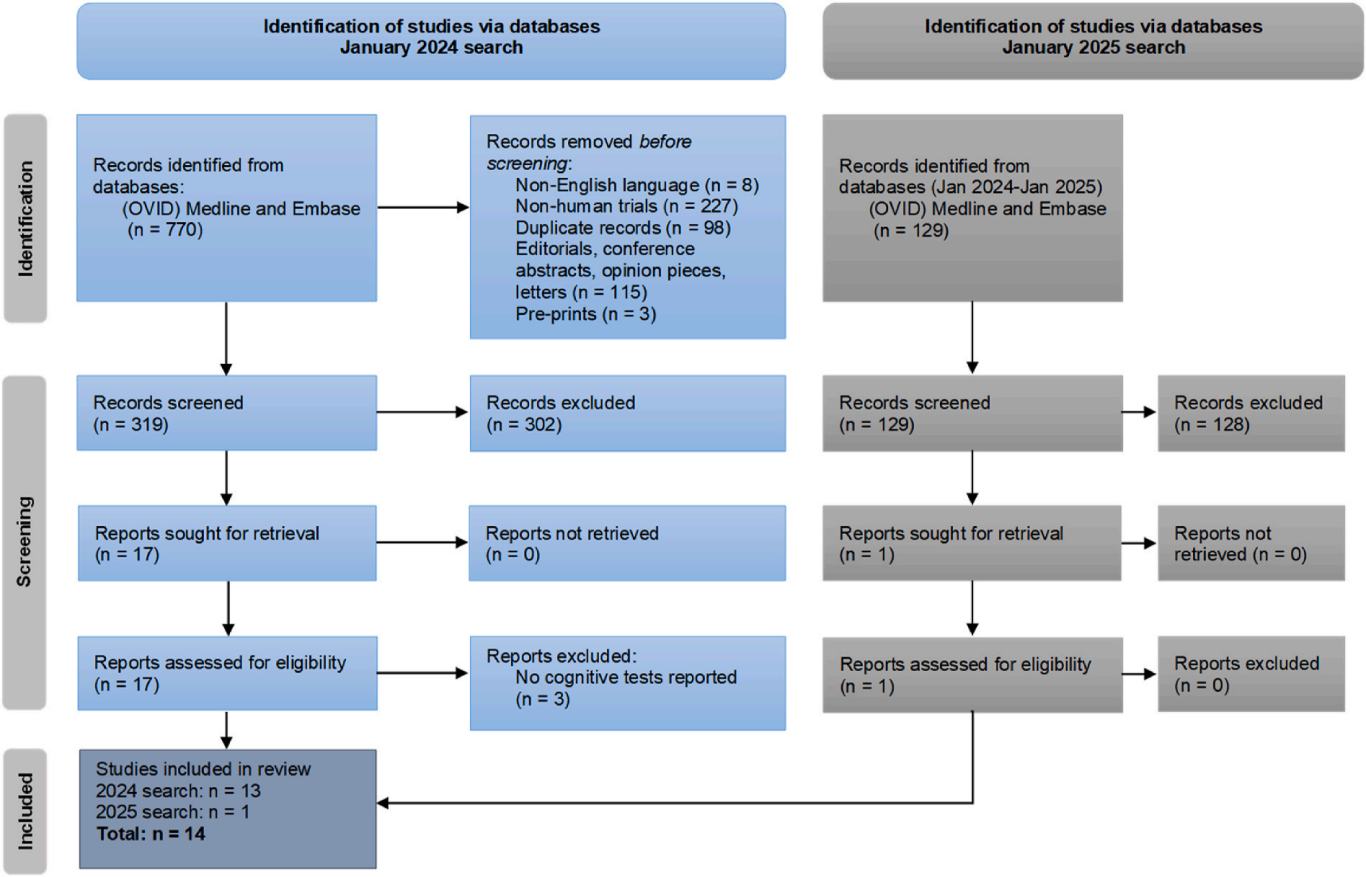
PRISMA diagram of the reviewing process.

*Note.* Adapted from Moher, Liberati (Moher et al., 2009).

**Table:** 

FMT search terms	“FMT” or “F?cal Microbiota Transplant∗” or “microbio∗ transfer” or “microbio∗ transplant∗” or “f?cal transplant∗” or “f?cal transfer”
Cognition search terms	“cognition” or “cognitive function” or “cognitive performance” or “cognitive abilities” or “cognitive” or “processing speed” or “executive function” or “learning” or “inhibition, psychological” or “exp memory” or “problem-based learning” or “problem solving” or “spatial learning” or “verbal learning” or “spatial navigation” or “decision making” or “cognitive dysfunction” or “dementia” or “memor∗” or “executive function∗” or “brain function”

## Data Availability

No data was used for the research described in the article.

## Appendix A

### Methods

Inclusion criteria were as follows: all patient groups, irrespective of gender, age, ethnicity, or disease; clinical trial on FMT, irrespective of dose or admission route; cognitive function as a primary or secondary outcome; English language; peer-reviewed (exclusion of pre-prints, editorials, opinion pieces and conference abstracts). Preclinical trials and reviews, systematic reviews, and meta-analysis were excluded (see Fig. 1).

Systematic searches were conducted in Medline and Embase (Ovid) on the January 31, 2024. Titles and abstracts were screened for meeting the inclusion criteria by one author (SA). Duplicates were removed manually. Full texts were reviewed to confirm eligibility. To capture newly published studies, a follow-up search was conducted in January 2025, restricted to publications from 2024 onward. Due to the small number and heterogeneity of studies, we conducted a narrative synthesis of findings. The review was not registered in PROSPERO due to time constraints. Ethical approval was not required for this review. This review follows a narrative review format with a systematic search strategy, appropriate for synthesising early clinical evidence in an emerging field.

### Results

The keyword search led to 770 results. After removal of duplicates and papers not matching the inclusion criteria, 319 studies were selected for screening. 17 abstracts matched the inclusion criteria. Full text reviews showed that four studies did not include cognitive measures in form of neuropsychological test scores and were therefore excluded. Full texts for the remaining 13 papers were reviewed and data on FMT administration, study population, design, cognitive domain tested and neuropsychological test utilised was extracted (see Table A.1, Appendix B). The follow-up search in January 2025 yielded 115 additional records, of which one met the inclusion criteria and was incorporated into the review, resulting in 14 studies included in this review.

### Systematic Search Strategy

Databases: Ovid Medline, Ovid Embase.
